# Surgical Management of Tracheostomy in Patients with Severe Burns and Cervical Involvement: Impact on Cervical Wound-Healing Disturbance

**DOI:** 10.3390/ebj7010016

**Published:** 2026-03-10

**Authors:** Julien-Moritz Thielmann, Wolfram Heitzmann, Michael Ried, Jan-Philipp Stromps, Maria von Kohout, Paul Christian Fuchs, Till Markowiak

**Affiliations:** 1 Department of Plastic, Reconstructive, Hand and Burn Surgery, Cologne-Merheim Medical Center (CMMC), Witten/Herdecke University, Campus Cologne-Merheim, 51109 Köln, Germany; 2 Department of Thoracic Surgery, University Hospital Regensburg, 93053 Regensburg, Germany

**Keywords:** tracheostomy, cervical burns, wound-healing disturbances, split-thickness skin grafting

## Abstract

Background: Major cervical burns often require tracheostomy (TT); however, it remains unclear whether TT timing relative to cervical wound coverage (WC) and the choice of coverage modality affect peristomal wound healing. Methods: In this retrospective single-center cohort study, we included 48 adults with thermally induced cervical burns who underwent TT between 2015 and 2024 in a specialized burn ICU. Eleven patients died before decannulation and were excluded from the primary endpoint analysis. Cervical wound-healing disturbance (CWHD) as a primary endpoint was assessed in the remaining 37 patients, including 13 treated with advanced cervical dressings and 24 treated with split-thickness skin grafts (STSG). Results: CWHD occurred in 2/13 (15.4%) with advanced dressings and 11/24 (45.8%) after STSG. Within the grafted subgroup, complication rates did not differ significantly according to TT sequencing relative to WC, TT–WC interval, grafting technique (mesh vs. Meek), or tracheostoma closure method (all *p* > 0.05). In multivariable logistic regression, only cervical burn depth independently predicted CWHD. Conclusion: In patients with cervical burns requiring TT, burn depth/severity, rather than TT timing, grafting strategy, or closure technique, appears to drive peristomal wound complications, supporting individualized planning instead of rigid algorithms.

## 1. Introduction

Severe burn injuries frequently necessitate prolonged mechanical ventilation (MV), particularly when the head and neck are affected, due to airway edema, inhalation injury, increased airway compromise, respiratory infections, and the systemic inflammatory response [1]. Tracheostomy (TT), as a cornerstone of advanced airway management, plays a pivotal role in these patients by facilitating pulmonary hygiene, reducing ventilator-associated complications, and potentially expediting weaning from MV [2,3].

When burns involve the anterior neck, the placement and subsequent management of the tracheostoma become surgically complex. The cervical region is anatomically delicate and functionally critical. Deep partial- or full-thickness burns in this area increase the risk of infection, limit reconstructive options, and impair wound healing. In these patients, tracheostomies often need to be performed through inflamed or regenerating tissue, which can interfere with graft integration and increase the likelihood of local complications.

Patients with deep cervical burn injuries represent a distinct and challenging subgroup due to the delicate anatomy and critical functional importance of the neck [4,5,6]. In these patients, TT intersects not only with airway management but also with reconstructive planning and wound healing. TT may be performed through partially debrided tissue, necrotic tissue following escharotomy, or simultaneously with surgical WC. Performing TT without simultaneous WC may allow for initial stabilization of the airway and easier surgical access, but it carries a risk of interfering with subsequent graft integration and local wound healing. Conversely, combining TT with WC in a single operative session may protect graft viability and reduce local complications, though it can increase procedural complexity and requires careful coordination. These considerations illustrate the surgical and reconstructive challenges unique to cervical burns and frame the rationale for investigating the impact of TT timing and WC on local wound healing outcomes.

Despite their clinical relevance, the optimal sequencing and timing of TT relative to cervical WC with autologous split-thickness skin grafts—whether applied as mesh or Meek grafts—remain undefined. Across critical care cohorts, “early” tracheostomy is commonly defined as placement within approximately 5 to 7 days after intubation or injury, whereas “delayed” tracheostomy is performed later; whether such timing concepts translate to cervical burn reconstruction is uncertain and likely context-dependent. Beyond burn depth as a non-modifiable driver of healing, modifiable aspects of TT management (sequencing relative to WC, TT–WC interval, closure strategy, and the choice of temporary dressings vs. definitive grafting) remain insufficiently studied in patients with anterior neck burns.

Additionally, approaches to tracheostoma closure, ranging from formal surgical revision to conservative management with occlusive dressings under the guidance of speech-language pathologists, vary considerably between centers and remain largely non-standardized [7,8]. Surgical closure, while offering the potential for more predictable anatomical closure, may pose increased risks of wound dehiscence or compromised graft integration in fragile, previously grafted tissue. Conversely, conservative management—such as gradual closure through occlusive dressings, often overseen by speech-language pathologists—may be less invasive but relies heavily on local tissue viability and patient compliance. These strategies can hypothetically influence scar quality, infection rates, and long-term functional outcomes, particularly in a region where contractures can significantly impact mobility and airway dynamics [9]. Despite their clinical relevance, techniques for tracheostoma management and closure have not been systematically evaluated and remain highly inconsistent across centers.

Inter-center heterogeneity in the timing and frequency of TT has been well documented across ICU populations, with studies demonstrating wide variation in procedural rates and timing definitions between institutions and research cohorts [10]. This marked heterogeneity underscores the lack of consensus-based pathways and suggests that decision-making in more complex scenarios such as tracheostoma management in patients with cervical burns is even less standardized and currently unsupported by outcome-driven evidence.

In light of these gaps, this study investigates the relationship between TT timing (relative to WC), the sequence of intervention, WC techniques, and stoma closure strategies in patients with cervical burns. By focusing on a surgically challenging and underrepresented subgroup, we aim to clarify which modifiable management decisions are associated with peristomal cervical wound-healing disturbance (CWHD) and which factors (such as burn depth) dominate outcomes.

## 2. Materials and Methods

### 2.1. Study Population

This retrospective single-center cohort study included adult patients (≥18 years) admitted to the burn intensive care unit of Cologne-Merheim Medical Center (CMMC) between January 2015 and June 2024 with thermally induced partial- (grade 2a and 2b) or full-thickness (grade 3) burns involving the anterior neck who underwent TT during intensive care treatment. Burn depth was assessed clinically by an attending burn surgeon based on standardized bedside criteria (capillary refill, sensibility, color, and bleeding characteristics) and, when excision was performed, confirmed intraoperatively according to tissue viability. Patients who died before decannulation or had insufficient clinical or operative documentation were excluded from analyses of the primary endpoint (CWHD).

TT was indicated in the context of anticipated prolonged MV or threatened airway compromise due to inhalation injury, progressive edema, or anatomical distortion. Whenever clinically feasible, TT was planned and performed electively, with an attempt at extubation prior to TT; however, in cases of clinically unstable or unsafe airways, where the attending anesthesiologist and burn surgeon jointly judged safe extubation unlikely or airway protection inadequate, TT was performed without delay.

Cervical WC strategies were selected according to burn depth, tissue viability, timing of definitive debridement, and institutional treatment protocols. Depending on these factors, wounds were managed either with temporary biosynthetic or bioactive dressings or with definitive autologous split-thickness skin grafts applied as mesh or Meek grafts. These bioactive and synthetic materials were primarily applied in superficial burns or during wound bed optimization, offering benefits such as moist healing, reduced pain, and decreased infection risk [11,12,13]. The study was approved by the local institutional ethics committee (S-27/2025) and conducted in accordance with the Declaration of Helsinki.

### 2.2. Data Acquisition

Demographic and clinical data were retrospectively collected from electronic health records, operative reports, and daily inpatient documentation. Extracted parameters included demographic characteristics, TBSA involvement, burn severity quantified by the Abbreviated Burn Severity Index (ABSI) score, and burn depth and distribution with explicit identification of involvement of the anterior cervical region. To assess the influence of surgical strategy on wound healing, detailed operative characteristics were recorded. These included TT and WC timing, sequencing of TT and cervical WC procedures (TT before WC, TT after WC, or performed simultaneously), TT closure technique, and the type of wound dressing or autologous grafting modality used (mesh graft or Meek graft), including donor site requirements. The cumulative number of surgical procedures and number of surgical procedures specifically targeting the cervical region were also recorded. The primary endpoint was CWHD in the peristomal region. CWHD was defined as a wound-healing disturbance that required operative re-coverage with autologous grafting or re-grafting based on the clinical judgment of an attending burn surgeon. It was typically characterized by a persistent non-epithelialized peristomal defect exceeding ap-proximately 4 × 4 cm at around 3 weeks after the most recent coverage procedure, usually in the setting of compromised graft take and/or ongoing tissue breakdown.

### 2.3. Study Endpoints

The primary endpoint was CWHD in the peristomal region requiring operative intervention with autologous grafting or re-grafting. Secondary endpoints included the number of dedicated cervical reconstructive procedures and associations between modifiable management factors—TT/WC sequencing, TT–WC interval, WC modality (advanced dressings vs. STSG), and tracheostoma closure strategy (surgical vs. conservative)—and the occurrence of CWHD.

### 2.4. TT Timing, Skin Grafting, and TT Management

The indication, timing, and technical approach to TT followed an individualized, case-based strategy. Decisions were made jointly by the burn surgery and intensive care teams after comprehensive assessment of airway safety, hemodynamic stability, and the anticipated duration of MV. When the airway could be safely maintained, TT was generally postponed until completion of the acute resuscitation phase and scheduled electively under optimal operative conditions. In patients with progressive airway edema, repeated failed extubation, or evolving respiratory compromise, earlier TT was favored to avoid emergent airway loss. Cervical anatomy, including edema and wound involvement, further influenced both the feasibility and technical conduct of TT. The temporal relationship between TT and cervical WC was likewise individualized. In some patients, TT was performed before WC to secure the airway in the setting of extensive edema or expected prolonged ventilation. In others, TT and autologous skin grafting were combined in a single session to minimize the number of procedures. When early grafting was feasible and airway compromise could be managed with translaryngeal intubation, TT was deferred until after initial WC with the aim of protecting the graft from peristomal contamination and mechanical stress.

For definitive reconstruction, the choice between mesh and Meek split-thickness skin grafting was guided by defect size and geometry, total body surface area (TBSA) involvement, and donor site availability. The Meek technique was preferred in patients with extensive burns or limited donor sites because of its higher expansion ratios and reduced donor morbidity, whereas mesh grafting was used for smaller or less extensive cervical defects. Tracheostoma closure was managed either conservatively or surgically depending on stoma size, local tissue quality, and overall clinical status. Conservative closure using occlusive taping or dressings was chosen when spontaneous epithelialization was expected to be reliable, while surgical revision was reserved for large, persistent, or unstable stomas, or when tension, infection risk, or functional concerns warranted operative repair. All key decisions on TT timing, grafting strategy, and stoma closure were made in a multidisciplinary setting.

### 2.5. Tracheostomy Technique

**Figure 1:**
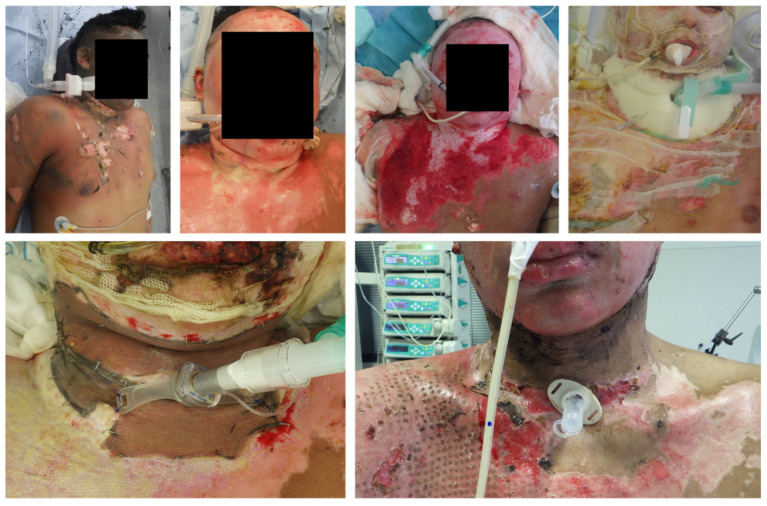
Cervical burn WC via STSG in Meek technique in proximity to a surgical TT.

TT remains a cornerstone in the airway management of patients with extensive burn injuries requiring prolonged mechanical ventilation or anticipated airway compromise [4,14,15,16]. In the context of cervical burns, where tissue integrity, vascular anatomy, and subsequent reconstructive strategies add complexity, the technical approach should be tailored with meticulous attention to anatomical and procedural variables. Surgical TT is predominantly performed in our clinic, as it provides a reliably stable airway, which is of paramount importance in our patient cohort during extensive dressing changes and mobilization. The technique usually entails a horizontal 3 cm skin incision placed half way between the inferior border of the cricoid cartilage and the sternal notch [10]. Dissection continues through subcutaneous tissue under direct visualization [17]. Particular care is taken to avoid injury to major vascular structures anterior to the trachea, especially the brachiocephalic artery, as well as possible anatomical variants such as a thyroidea artery or prominent brachiocephalic veins [18,19,20,21]. Intraoperative identification and potential division of the thyroid isthmus are frequently necessary to ensure unimpeded access to the tracheal rings [10]. The tracheal window is typically fashioned between the second and third rings, allowing for direct insertion of a cuffed TT cannula [17]. In burn patients, surgical TT offers distinct advantages: improved exposure in the presence of distorted or fibrotic soft tissue planes, superior control of perioperative bleeding, and the option to construct a stable epithelialized stoma when prolonged cannulation is anticipated [15]. Although surgical TT is not without risk, the procedure offers superior safety in scenarios where cervical anatomy is distorted or contaminated, and is therefore preferred in our burn patients requiring concurrent or impending neck WC. These considerations are particularly relevant when reconstructive interventions to the neck are planned or ongoing. Representative clinical images illustrating the anatomical relationship between the TT stoma and the cervical burn wound before and after WC with autologous grafts, including examples of peristomal wound healing disturbance requiring re-grafting (Figure 1).

Percutaneous dilatational tracheostomy (PDT), though widely adopted in intensive care units for its procedural efficiency and lower immediate surgical burden, presents notable limitations in the setting of cervical burns [15,22]. PDT involves a Seldinger-guided approach, wherein an introducer needle is advanced into the tracheal lumen typically between the second and fourth rings under bronchoscopic guidance [22,23]. A guidewire is then placed, over which serial dilators are passed to create a tract sufficient for tube placement. Real-time endoscopic monitoring reduces the risk of posterior wall injury and ensures intratracheal positioning [22,24].

Despite its minimally invasive profile, PDT is often contraindicated in patients with distorted cervical anatomy, extensive soft tissue injury, or anticipated reconstructive procedures [23]. Its dependence on intact cervical soft-tissue planes makes PDT suboptimal in the presence of tissue destruction, edema, or local contamination.

In this cohort, the selection between surgical and PDT was not governed by a fixed institutional protocol but rather reflected a case-by-case assessment, incorporating anatomical feasibility, urgency of airway control, extent of cervical injury, and interdisciplinary consensus between critical care, burn surgery, and anesthesiology teams.

### 2.6. Statistical Analysis

All statistical analyses were performed using IBM SPSS Statistics, Version 29.0 (IBM Corp., Armonk, NY, USA). Categorical variables—including autologous grafting technique (mesh vs. Meek), TT/WC sequencing (TT before, after, or simultaneous with WC), and tracheostoma closure method (conservative vs. surgical)—were reported as frequencies and percentages. The interval between TT and WC was treated as a continuous variable. Continuous variables, such as age, TBSA (%), ABSI score, and interval between TT and WC, were tested for normality using the Shapiro–Wilk test and presented as mean ± standard deviation (SD) for normally distributed data or median with interquartile range (IQR) for non-normally distributed data. Differences in continuous baseline characteristics across cervical coverage modalities (advanced dressings, mesh grafting, Meek grafting) were assessed using one-way analysis of variance (ANOVA) or Kruskal–Wallis as appropriate. For multivariable analysis, we constructed a binary logistic regression model with impaired cervical wound healing as the dependent variable and included TBSA, ABSI score, cervical burn depth (grade 2a, 2b, 3°), coverage modality (advanced dressings, mesh, Meek), infection, and pneumonia as covariates. Associations between grafting technique, TT/WC sequencing, interval between TT and WC, tracheostoma closure method, and the primary endpoint of CWHD were assessed using Pearson’s chi-squared test or Fisher’s exact test for categorical variables and independent samples *t*-test or Mann–Whitney U test for continuous variables as appropriate. A two-sided *p*-value < 0.05 was considered statistically significant.

## 3. Results

### 3.1. Demographics

**Figure 2:**
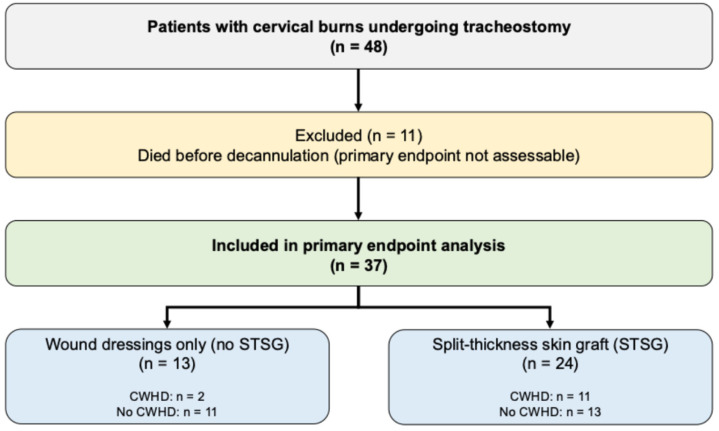
Study flow and subgroup allocation.

A total of 48 adult patients with thermally induced cervical burns requiring TT were included in the study cohort. Eleven patients died before decannulation and were therefore excluded from analyses of the primary endpoint, leaving 37 patients for wound outcome assessment. The mean age at time of injury was 48.1 ± 17.7 years, and the majority of patients were male. Baseline characteristics for the full cohort (n = 48) are summarized in Table 1 and Figure 2.

The median number of dedicated reconstructive procedures per patient targeting the cervical region was one (IQR 1), while the total number of surgical interventions across all anatomical sites averaged 6.1 ±3.2 per patient. With regard to burn depth at the cervical site, 14 patients (29.2%) presented with superficial partial-thickness burns (grade 2a), 14 (29.2%) with deep partial-thickness burns (grade 2b), and 20 (41.7%) with full-thickness (grade 3) injuries.

Analysis of the procedural sequencing revealed that in 31 patients (64.6%), TT was performed either concomitantly with or subsequent to cervical WC, while in 17 cases (35.4%) the airway was surgically secured prior to definitive reconstruction. Regarding reconstructive modality, 16 patients (33.3%) were managed with advanced dressings alone, while 17 (35.4%) underwent cervical mesh graft transplantation and 15 (31.3%) received Meek grafts.

### 3.2. Clinical Data

All patients underwent TT during their clinical course. The vast majority (n = 46; 95.8%) were managed via open surgical TT, whereas percutaneous techniques were used in only two cases (4.2%). The median interval from burn injury to TT placement was 6 days (IQR 9). Decannulation was achieved at a median of 32 days post-tracheostomy (IQR 39). With regard to surgical sequencing, TT was performed prior to cervical wound reconstruction in 35.4% of patients (n = 17), whereas in the majority (n = 31, 64.6%) it was placed after or concomitantly with surgical coverage. Patients underwent a mean of 6.1 (±3.2) surgical procedures during their hospital stay, consistent with the need for staged debridement, wound bed preparation, and definitive soft tissue coverage. The median number of plastic coverings was 1 (IQR 1), though some patients required multiple revisions due to local infection, mechanical shear or insufficient graft take. Three principal strategies were employed for cervical WC. Advanced wound dressings such as Dressilk© and Suprathel© were used in 33.3% (n = 16) of cases. Definitive surgical reconstruction was achieved either by mesh grafting (n = 17, 35.4%) or via Meek grafting (n = 15, 31.3%). Clinical data are displayed in Table 1.

Age and ABSI score were comparable across coverage groups (*p* = 0.77 and *p* = 0.15). In contrast, total burned body surface area differed significantly, with the highest values observed in patients undergoing Meek grafting (*p* = 0.02). Coverage modality was also strongly associated with sex and cervical burn depth: Meek grafting was performed exclusively in male patients, whereas the proportion of females was highest in the mesh group (*p* = 0.009). Advanced wound dressings were predominantly used for superficial dermal (2a) cervical burns, while mesh grafts were almost exclusively applied in full-thickness injuries and Meek grafts mainly in deep dermal (2b) burns (*p* < 0.001). The baseline characteristics stratified by cervical coverage modality are presented in Table 2.

### 3.3. Wound Healing Outcomes in Relation to Surgical Timing and Technique

In the analyzed cohort surviving to decannulation (n = 37), CWHD requiring repeat grafting occurred in 13 patients (35.1%). In the subgroup managed exclusively with biosynthetic cervical dressings (n = 13), CWHD occurred in 2 cases (15.4%). Among patients who underwent definitive cervical WC using STSG and survived to decannulation (n = 24), CWHD was observed in 11 cases (45.8%). When stratified by TT timing relative to cervical WC within the grafted subgroup, 8/16 patients (50.0%) who received TT after cervical WC developed CWHD, compared with 3/8 (37.5%) in whom TT preceded or was concomitant with WC (*p* = 0.56).

To further assess the impact of timing, the cohort was subdivided based on the interval between TT and surgical WC. Among patients with a time interval of fewer than five days (n = 12), impaired healing occurred in 4 cases (33.3%). In comparison, 7 patients (58.3%) in the group with an interval of five days or more (n = 12) experienced wound healing disturbances, although this difference did not reach statistical significance (*p* = 0.22).

**Figure 3:**
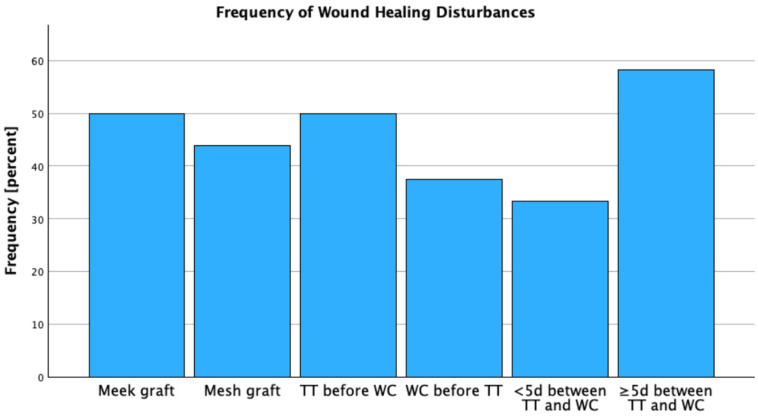
Frequency of Wound Healing Disturbances.

With respect to the reconstructive modality, 7 of 16 patients (43.8%) treated with mesh grafts and 4 of 8 patients (50.0%) treated using Meek grafting exhibited complications in local wound healing (*p* = 0.22). Cervical wound healing disturbances were observed in 11 patients (45.8%). Among patients receiving surgical tracheostoma closure, 4 of 10 (40%) experienced wound healing complications, compared with 7 of 14 (50%) in those managed with conservative occlusive closure, with no statistically significant difference between groups (*p* = 0.63). The frequency of CWHD stratified by grafting technique, TT/WC sequencing, and TT closure method is displayed in Table 3 and Figure 3.

### 3.4. Multivariable Logistic Regression Model

To identify independent predictors of impaired cervical wound healing, we constructed a multivariable logistic regression model including TBSA, ABSI score, cervical burn depth (grade 2a, 2b, 3), WC modality (advanced dressings, mesh and Meek grafting), infection, and pneumonia as covariates. In this model, cervical burn depth was the only variable independently associated with wound-healing disturbance. Compared with superficial partial-thickness (grade 2a) burns, deep partial-thickness burns (grade 2b) were associated with a markedly increased odds of impaired healing (odds ratio [OR] 26.8, 95% CI 1.0–702.1; *p* = 0.049), and full-thickness (grade 3) burns showed an even higher odds (OR 132.5, 95% CI 2.7–6575.2; *p* = 0.014). In contrast, TBSA (*p* = 0.36), ABSI score (*p* = 0.36), coverage modality (mesh vs. advanced dressings: *p* = 0.21; Meek vs. advanced dressings: *p* = 0.40), infection (*p* = 0.999), and pneumonia (*p* = 0.41) were not independently associated with cervical wound-healing disturbance in this cohort.

### 3.5. Tracheostomy Timing Relative to Burn Injury

In the evaluable cohort (excluding patients who died before decannulation/closure), the interval between burn injury and tracheostomy was similar between patients with and without CWHD. Patients without CWHD had a median of 7 days (IQR 10), whereas patients with CWHD had a median of 4 days (IQR 7); this difference was not statistically significant in Mann–Whitney U test (*p* = 0.16). When dichotomized using a 5-day cut-off, CWHD occurred in 7/18 (38.9%) patients with early tracheostomy (≤5 days) and in 6/19 (31.6%) patients with delayed tracheostomy (>5 days), with no significant association (Fisher’s exact test; *p* = 0.74).

## 4. Discussion

This retrospective cohort study examined how TT timing and cervical burn wound treatment intersect in the management of severely burned patients, with a particular focus on potentially modifiable factors at the interface of airway security and cervical reconstruction. Because almost all tracheostomies in our cohort were performed as open surgical procedures (95.8%), comparative conclusions regarding percutaneous dilatational techniques are limited; nonetheless, the potential for different peristomal contamination and infection profiles between approaches should be considered when extrapolating our findings.

Clinically, an “early” tracheostomy may facilitate earlier sedation reduction, pulmonary toilet and weaning, and it can ease oral and facial wound care; potential drawbacks include performing TT through edematous or evolving cervical tissue and creating a stoma before definitive debridement and reconstruction. Conversely, a “delayed” tracheostomy may allow clearer demarcation and debridement planning, but prolonged translaryngeal intubation and later tracheostoma creation can complicate cervical graft protection and may coincide with an increasing peristomal microbial burden. In our cohort, the burn-to-tracheostomy interval was not significantly associated with CWHD (median 7 versus 4 days; *p* = 0.16), and CWHD rates were comparable for ≤5 versus >5 days (*p* = 0.74).

Previous studies have predominantly focused on TT timing in critically ill or trauma patients, often neglecting the unique challenges faced in the context of extensive burns involving the neck [6,25,26,27,28]. In this anatomical region, the dual burden of respiratory compromise and complex wound care introduces conflicting priorities: on the one hand, early airway protection via TT may reduce ventilator dependence and facilitate secretion management; on the other hand, such interventions may interfere with local wound healing, particularly when performed in proximity to skin grafts or dressings applied during cervical WC. Large randomized trials and meta-analyses in mixed ICU populations suggest that early TT can shorten MV and ICU stay, while effects on mortality and ventilator-associated pneumonia remain modest or inconsistent, highlighting that timing decisions often balance systemic benefits against local risks in anatomically vulnerable regions such as the neck [11,27].

No statistically significant difference in wound complication rates was observed between patients undergoing TT before, during, or after cervical WC. Although CWHD was numerically higher when coverage occurred before TT or simultaneously (50.0%) compared with TT preceding coverage (37.5%), this did not reach statistical significance. Importantly, both scenarios expose the wound or the freshly transplanted skin to TT-related stressors, such as secretions and chronically increased local moisture levels, bacterial load, cannula movement, and cuff pressure. The pathophysiological basis for potential interactions remains plausible. These factors may impair revascularization and epithelialization, regardless of the surgical sequence, particularly in the early postoperative period. Thus, our findings suggest that the timing alone may be less decisive than surgical technique, local hygiene measures, and graft stabilization strategies in determining wound outcomes. Nevertheless, given the small sample size and heterogeneity in perioperative protocols, these trends should be interpreted with caution. Similar observations have been reported in larger burn cohorts, where TT was more common in patients with higher TBSA and ABSI scores but did not independently increase local complication rates or mortality when contemporary airway and wound care strategies were implemented [15,27].

Interestingly, the interval between TT and cervical WC also did not result in a statistically significant difference in CWHD rates. While longer intervals (≥5 days) were associated with numerically higher disturbance rates, the cohort size limits definitive inference. Importantly, the median duration from TT placement to decannulation in our cohort was 32 days, a timeframe in which tracheostomy tubes may become increasingly colonized and peristomal granulation may develop. Prospective data suggest that granulation and microbial burden rise when tubes remain in situ beyond one month, supporting monthly tube exchange in long-term cannulation settings [29].

The WC technique emerged as another potential factor influencing outcomes. Patients treated with mesh grafts demonstrated a lower incidence of CWHD compared to those undergoing Meek grafting, although this difference was not statistically significant. Beyond autologous grafting and temporary biosynthetic dressings, dermal substitutes (e.g., Integra^®^, BTM) have been proposed for complex cervical wounds and may offer a staged option for wound bed preparation and subsequent grafting; however, concurrent use with TT was not systematically applied in our cohort and could not be evaluated. Nevertheless, this strategy may be worth exploring in future protocols, particularly when peristomal shear, contamination or repeated graft loss are anticipated [30].

Approaches to tracheostoma closure range from formal surgical closure to conservative, occlusive management. Although our cohort did not show statistically significant differences in CWHD between closure approaches, this finding should be interpreted cautiously. From a clinical standpoint, surgical closure may shorten the period of exposure, potentially reducing contamination, infection risk and functional discomfort; it may also mitigate invagination or hypertrophic scarring by enabling layered closure and contour control. Conversely, conservative closure can be attractive in fragile, previously grafted tissue or in medically unstable patients, but it may prolong local moisture and shear and requires careful monitoring. Future studies should therefore incorporate patient-reported comfort, scar quality and functional outcomes when comparing closure strategies [31].

The cervical region is particularly vulnerable to wound healing disturbances due to its mobility, thin skin, and critical functional roles in respiration, swallowing and neck motion. Clinical data have shown that the extreme mobility of the neck predisposes it to contracture formation post-burn, and that limited early pressure therapy or suboptimal positioning is associated with a higher need for reconstructive intervention [32,33]. Moreover, mechanical stresses from patient positioning, coughing, or airway suctioning may contribute to micro-shear forces that disrupt fragile neovascularization in early graft stages. These pathophysiological aspects emphasize the need for meticulous perioperative planning, including secure graft fixation, optimized dressing techniques, and protective measures against mechanical disruption. This is consistent with series reporting high rates of post-burn cervical contracture and improved outcomes with early splinting, positioning, and pressure therapy, underlining the importance of rigorous rehabilitation protocols alongside surgical decision-making [32].

While our analysis did not demonstrate statistically significant differences in wound healing outcomes between sequential and simultaneous procedures, the lack of an observable penalty for simultaneous TT and WC supports our clinical tendency to perform both interventions in a single session. This approach minimizes the need for a second operation, reduces cumulative anesthetic exposure, and streamlines perioperative coordination without compromising graft integrity. The absence of significant differences in wound outcomes across grafting techniques, TT closure strategies, and the timing relationship between TT and WC suggests that no single procedural pathway is inherently superior for preventing cervical wound complications. Rather than relying on predefined sequencing algorithms, the data support a patient-tailored strategy in which procedural timing and technique are adapted to the individual wound environment and airway requirements. This approach depends on close collaboration between burn surgeons, intensivists, and wound care teams to balance airway security with the conditions needed for reliable graft integration and infection control.

Future research should include prospective, multicenter studies designed to evaluate standardized protocols, incorporating variables such as graft type, postoperative immobilization strategies, and objective measures of wound healing (e.g., time to epithelialization, graft loss percentage, infection rates). Longitudinal outcomes, including scar quality, cervical mobility, and reconstructive requirements, should also be evaluated to determine whether early procedural decisions influence long-term function and aesthetic results.

## 5. Conclusions

This study highlights the complexity of coordinating TT and cervical burn WC. In our cohort, burn depth/severity emerged as the dominant predictor of peristomal CWHD, whereas TT timing relative to WC, grafting technique, and closure strategy were not significantly associated with CWHD within the analyzed STSG subgroup. These findings support meticulous, individualized planning that balances airway security with reconstructive principles rather than relying on rigid timing algorithms.

## 6. Limitations

From a methodological standpoint, several limitations merit discussion. The retrospective nature of our data collection, together with the relatively small sample size, the single-center design, and the limited number of percutaneous tracheostomies inherently restrict statistical power and generalizability. In addition, the cohort was comparatively young and had a relatively low BMI distribution, which may limit extrapolation to older or obese burn populations with different wound-healing dynamics. Burn depth was recorded in routine clinical practice and, although assessed by experienced burn surgeons (and confirmed intraoperatively when excision was performed), misclassification cannot be fully excluded. Finally, CWHD was defined pragmatically by the need for repeat grafting, and variables such as microbial colonization, shear forces, and detailed scar outcomes were not quantified.

In addition to wound-specific outcomes, broader implications such as scar quality, cervical mobility, and the need for secondary reconstructive interventions should be explored. Given the esthetic and functional significance of the neck, even minor wound complications can translate into long-term morbidity, including hypertrophic scarring, contractures, and reduced quality of life. A clearer understanding of how TT timing affects these downstream outcomes could inform both acute and rehabilitative care strategies.

## Figures and Tables

**Table 1:** Demographics and clinical data of the full study cohort (n = 48).

	All Patients n = 48
**Demographics**	
Age, mean ± SD [years]	48.1 ± 17.7
Male, n (%)	36 (75.0)
BMI, median (IQR) [kg/m^2^]	26.1 (5.76)
Affected TBSA, mean ± SD [%]	28.0 ± 16.9
ABSI Score, median (IQR)	8 (5)
Cervical burn depth, n (%)	
- Superficial partial-thickness (2a)	14 (29.2)
- Deep partial-thickness (2b)	14 (29.2)
- Full-thickness (3)	20 (41.7)
**Clinical Data**	
TT Performed, n (%)	48 (100)
- Surgical	46 (95.8)
- Percutaneous	2 (4.2)
TT Timing, days post-burn (median, IQR)	6 (9)
Time TT to decannulation, days (median, IQR)	32 (39)
Sequence TT relative to cervical reconstruction, n (%)	
- TT before coverage	17 (35.4)
- Coverage before/simultaneous to TT	31 (64.6)
Total operations (mean ± SD)	6.1 ± 3.2
Cervical reconstructive procedures (median, IQR)	1 (1)
Coverage method, n (%)	
- Advanced dressings	16 (33.3)
- Mesh grafts	17 (35.4)
- Meek grafts	15 (31.3)

ABSI: Abbreviated Burn Severity Index, BMI: Body Mass Index, IQR: Interquartile Range, SD: Standard Deviation, TBSA: Total Body Surface Area Burned, TT: Tracheostomy.

**Table 2:** Baseline characteristics by cervical coverage modality in the full cohort (n = 48).

	Wound Dressings (n = 16)	Mesh Graft (n = 17)	Meek Graft (n = 15)	*p*-Value
Age, years, mean ± SD	51.4 ± 18.1	50.4 ± 19.3	46.6 ± 20.2	0.77
Burned TBSA, %, mean ± SD	33.1 ± 20.9	26.1 ± 15.3	46.8 ± 24.1	0.02
ABSI score, median (IQR)	7 (5.5)	7 (3.5)	9 (2.5)	0.15
Male sex, n (%)	12 (75.0)	9 (52.9)	15 (100.0)	0.009 *
Cervical burn depth, n (%)				
- Superficial partial (2a)	13 (81.3)	0 (0.0)	1 (6.7)	
- Deep partial (2b)	3 (18.8)	3 (17.6)	8 (53.3)	<0.001 *
- Full-thickness (3)	0 (0.0)	14 (82.4)	6 (40.0)	

ABSI: Abbreviated Burn Severity Index, IQR: Interquartile range, TBSA: Total body surface area burned, SD: Standard Deviation. * Statistically significant.

**Table 3:** CWHD in patients treated with split-thickness skin grafting (STSG) who survived to decannulation (n = 24).

	All Patients (n = 24)	TT After WC (n = 16)	TT Before WC or Simultaneously (n = 8)	*p*-Value
Number of cervical reconstructive procedures, median (IQR)	1 (2)	1 (2)	1.5 (2)	0.61
Wound healing disturbances, n (%)	11 (45.8)	8 (50)	3 (38)	0.56
	**All patients (n = 24)**	**WC Meek (n = 8)**	**WC Mesh (n = 16)**	***p*** **-value**
Number of cervical reconstructive procedures, median (IQR)	1 (2)	1.5 (2)	1 (2)	1.0
Wound healing disturbances, n (%)	11 (45.8)	4 (50)	7 (44)	0.22
	**All patients (n = 24)**	**<5 days between TT and WC** **(n = 12)**	**≥5 days between TT and WC (n = 12)**	***p*** **-value**
Number of cervical reconstructive procedures, median (IQR)	1 (2)	1 (1)	2 (2)	0.22
Wound healing disturbances, n (%)	11 (45.8)	4 (33)	7 (58)	0.22
	**All patients (n = 24)**	**Surgical closure** **(n = 10)**	**Non-surgical** **closure (n = 14)**	***p*** **-value**
Number of cervical reconstructive procedures, median (IQR)	1 (2)	1 (2)	1.5 (2)	0.88
Wound healing disturbances, n (%)	11 (45.8)	4 (40)	7 (50)	0.63

IQR: Interquartile Range, STSG: split-thickness skin grafting, TT: Tracheostomy, WC: Wound coverage.

## Data Availability

The datasets supporting the conclusions of this article will be made available by the authors on request.

## Abbreviations

The following abbreviations are used in this manuscript:

ABSI	Abbreviated Burn Severity Index
ANOVA	one-way analysis of variance
BMI	Body Mass Index
CI	Confidence interval
CMMC	Cologne-Merheim Medical Center
CWHD	Cervical wound-healing disturbance
ICU	Intensive care unit
IQR	Interquartile Range
MV	Mechanical ventilation
OR	odds ratio
PDT	Percutaneous dilatational tracheostomy
SD	Standard Deviation
STSG	split-thickness skin grafting
TBSA	Total Body Surface Area Burned
TT	Tracheostomy
USA	United States of America
WC	Wound coverage

## References

[1] Giurgiu R.A., Bordeanu-Diaconescu E.M., Grosu-Bularda A., Frunza A., Grama S., Costache R.A., Cristescu C.-I., Neagu T.-P., Lascar I., Hariga C.-S. (2025). The Impact of Face and Neck Burns on Respiratory Complications and Mortality. Eur. Burn J..

[2] Saffle J.R., Morris S.E., Edelman L. (2002). Early Tracheostomy Does Not Improve Outcome in Burn Patients. J. Burn Care Rehabil..

[3] Ruiz S., Puyana S., McKenney M., Hai S., Mir H. (2023). Outcomes of Tracheostomy on Burn Inhalation Injury. Eplasty.

[4] Shah J.K., Stanton E.W., Najafali D., Nazerali R., Sheckter C.C. (2025). Early versus Late Tracheostomy in Critically Injured Burn Survivors: A National, Multi-Database Analysis. J. Burn Care Res..

[5] Tanaka A., Uchiyama A., Kitamura T., Sakaguchi R., Komukai S., Matsuyama T., Yoshida T., Tokuhira N., Iguchi N., Fujino Y. (2022). Association between Early Tracheostomy and Patient Outcomes in Critically Ill Patients on Mechanical Ventilation: A Multicenter Cohort Study. J. Intensive Care.

[6] Khammas A.H., Dawood M.R. (2018). Timing of Tracheostomy in Intensive Care Unit Patients. Int. Arch. Otorhinolaryngol..

[7] Mills C.S., Newman H., Iezzi C., Sutt A.L., Jones R., Sadiq J., Ginnelly A., Jones G., Obe S.W. (2023). Speech and Language Therapy Service Provision to UK Intensive Care Units: A National Survey. Adv. Commun. Swallowing.

[8] McGowan S.L., Ward E.C., Wall L.R., Shellshear L.R., Spurgin A.L. (2014). UK Survey of Clinical Consistency in Tracheostomy Management. Int. J. Lang. Commun. Disord..

[9] Sinha I., Nabi M., Simko L.C., Wolfe A.W., Wiechman S., Giatsidis G., Bharadia D., McMullen K., Gibran N., Kowalske K. (2019). Head and Neck Burns Are Associated with Long-Term Patient-Reported Dissatisfaction with Appearance: A Burn Model System National Database Study. Burns.

[10] Cheung N.H., Napolitano L.M. (2014). Tracheostomy: Epidemiology, Indications, Timing, Technique, and Outcomes. Respir. Care.

[11] Wang M., Li H., Luo Y., Chen J., Tang Z., Wei Y., Yang Q., Xiao W., You W., Feng M. (2025). Biomimetic Nano Dressing in Wound Healing: Design Strategies and Application. Burn. Trauma.

[12] Hundeshagen G., Collins V.N., Wurzer P., Sherman W., Voigt C.D., Cambiaso-Daniel J., Nunez-Lopez O., Sheaffer J., Herndon D.N., Finnerty C.C. (2018). A Prospective, Randomized, Controlled Trial Comparing the Outpatient Treatment of Pediatric and Adult Partial-Thickness Burns with Suprathel or Mepilex Ag. J. Burn Care Res..

[13] Madaghiele M., Demitri C., Sannino A., Ambrosio L. (2014). Polymeric Hydrogels for Burn Wound Care: Advanced Skin Wound Dressings and Regenerative Templates. Burn Trauma.

[14] Saquib S., Jesic L., Carroll J., Flores C., Chestovich P., Fraser D. (2023). The Role of a Tracheostomy in the Critically Ill Burn Patient. J. Burn Care Res..

[15] Smailes S.T., Ives M., Richardson P., Martin R.V., Dziewulski P. (2014). Percutaneous Dilational and Surgical Tracheostomy in Burn Patients: Incidence of Complications and Dysphagia. Burns.

[16] Mourelo M., Galeiras R., Pértega S., Freire D., López E., Broullón J., Campos E. (2015). Tracheostomy in the Management of Patients with Thermal Injuries. Indian J. Crit. Care Med..

[17] Muscat K., Bille A., Simo R. (2017). A Guide to Open Surgical Tracheostomy. Shanghai Chest.

[18] Weightman W.M., Gibbs N.M. (2018). Prevalence of Major Vessels Anterior to the Trachea at Sites of Potential Front-of-Neck Emergency Airway Access in Adults. Br. J. Anaesth..

[19] Joshi K.D., Singh A., Singh D.K., Gupta V., Saxena S., Hota A., Girdhar S., Singhavi H.R., Thapa S., Leelakanth K. (2025). Tracheo-Innominate Artery Fistula: A Systematic Review of Diagnostic and Management Strategies. Otolaryngol. Head Neck Surg..

[20] Totlis T., Natsis K., Achlatis V., Pettas T., Piagkou M. (2023). Thyroidea Ima Artery Multiple Branching Pattern over the Trachea. Surg. Radiol. Anat..

[21] Varelli G., Cioni R., Casagli S., Cervelli R., Brusasco C., Forfori F., Corradi F. (2019). Conservative Management of Trachea-to-Innominate Artery Transfixion with a Guidewire during Percutaneous Tracheostomy: A Case Report. BMC Anesthesiol..

[22] Ciaglia P., Graniero K.D. (1992). Percutaneous Dilatational Tracheostomy: Results and Long-Term Follow-Up. Chest.

[23] Ciaglia P., Firsching R., Syniec C. (1985). Elective Percutaneous Dilatational Tracheostomy: A New Simple Bedside Procedure; Preliminary Report. Chest.

[24] Cho Y.J. (2012). Percutaneous Dilatational Tracheostomy. Tuberc. Respir. Dis..

[25] Merola R., Iacovazzo C., Troise S., Marra A., Formichella A., Servillo G., Vargas M. (2024). Timing of Tracheostomy in ICU Patients: A Systematic Review and Meta-Analysis of Randomized Controlled Trials. Life.

[26] Meng L., Wang C., Li J., Zhang J. (2016). Early vs Late Tracheostomy in Critically Ill Patients: A Systematic Review and Meta-Analysis. Clin. Respir. J..

[27] Andriolo B.N., Andriolo R.B., Saconato H., Atallah Á.N., Valente O. (2015). Early versus Late Tracheostomy for Critically Ill Patients. Cochrane Database Syst. Rev..

[28] Koch T., Hecker B., Hecker A., Brenck F., Preu M., Schmelzer T., Padberg W., Weigand M.A., Klasen J. (2012). Early Tracheostomy Decreases Ventilation Time but Has No Impact on Mortality of Intensive Care Patients: A Randomized Study. Langenbecks Arch. Surg..

[29] Saravanam P.K., Jayagandhi S., Shajahan S. (2022). Microbial Profile in Tracheostomy Tube and Tracheostoma: A Prospective Study. Indian J. Otolaryngol. Head Neck Surg..

[30] He M., Choi J., Mir I., Al Wahaiby M., Papp A. (2025). Integra® versus NovoSorb® Biodegradable Temporizing Matrix for Burn Wounds: A Comparative Systematic Review and Meta-Analysis. Burns.

[31] Kao C.N., Liu Y.W., Chang P.C., Chou S.H., Lee S.S., Kuo Y.R., Huang S.H. (2020). Decision Algorithm and Surgical Strategies for Managing Tracheocutaneous Fistula. J. Thorac. Dis..

[32] Sharp P.A., Dougherty M.E., Kagan R.J. (2007). The Effect of Positioning Devices and Pressure Therapy on Outcome after Full-Thickness Burns of the Neck. J. Burn Care Res..

[33] Mody N.B., Bankar S.S., Patil A. (2014). Post Burn Contracture Neck: Clinical Profile and Management. J. Clin. Diagn. Res..

